# Atomistic understandings of reduced graphene oxide as an ultrathin-film nanoporous membrane for separations

**DOI:** 10.1038/ncomms9335

**Published:** 2015-09-23

**Authors:** Li-Chiang Lin, Jeffrey C. Grossman

**Affiliations:** 1 Department of Materials Science and Engineering, Massachusetts Institute of Technology, 77 Massachusetts Avenue, Cambridge, Massachusetts 02139, USA; 2 Department of Process and Energy, Delft University of Technology, Leeghwaterstraat 39, 2628 CB Delft, The Netherlands

## Abstract

The intrinsic defects in reduced graphene oxide (rGO) formed during reduction processes can act as nanopores, making rGO a promising ultrathin-film membrane candidate for separations. To assess the potential of rGO for such applications, molecular dynamics techniques are employed to understand the defect formation in rGO and their separation performance in water desalination and natural gas purification. We establish the relationship between rGO synthesis parameters and defect sizes, resulting in a potential means to control the size of nanopores in rGO. Furthermore, our results show that rGO membranes obtained under properly chosen synthesis conditions can achieve effective separations and provide significantly higher permeate fluxes than currently available membranes.

## Abstract (web summary)

Ultrathin-film nanoporous membranes promise low-cost and high-performance separation for applications such as water desalination and the purification of natural gas. Here, the authors adopt a molecular dynamics approach to assess the potential of reduced grapheme oxide as such a material.

## Graphical abstract

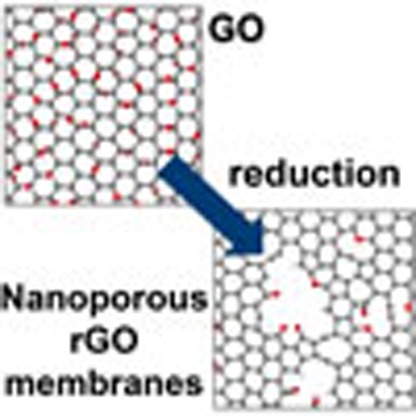

Membrane technologies are essential in many energy-related separation processes such as water desalination and gas treatments^1^,^2^. To achieve an effective separation process, membrane pores need to be sufficiently large to allow passage of one component but small enough to block other components from entering. As natural resources have become increasingly limited, reducing the energy consumption and capital cost of membrane separation processes is of utmost importance. In particular, to achieve such reductions, ultra-permeable membranes (that is, high permeate fluxes) have an important role to play^3^,^4^. As fluxes across a membrane scale inversely with the membrane's thickness, one can imagine that the ‘ultimate' membrane may be in the form of a one-atom-thick material with nanopores, which allows unprecedentedly high fluxes. A new paradigm in membrane science based upon nanoporous ultrathin-film membranes is therefore of great interest to push separation technologies forward significantly^5^,^6^,^7^.

Recently, one-atom-thick nanoporous graphene has drawn considerable interest for its potential in separation applications. Several computational studies have predicted that nanoporous graphene can provide a huge improvement in water permeability by as high as two to three orders of magnitude compared with commercially available reverse osmosis (RO) membranes^5^,^8^. Such an enhanced permeability is anticipated to substantially reduce the energy requirement and capital cost associated with RO desalination^4^. In addition, this novel class of materials has been shown to provide opportunities to improve the energy-efficiency of gas separation processes^5^,^6^,^7^,^9^. Although such exciting potential of nanoporous graphene in separations has been further demonstrated in the lab^10^,^11^, the large-scale manufacturability of nanoporous graphene membranes remains an open question and a great challenge. To date, advanced production techniques, including chemical vapor deposition (CVD) growth^12^ and roll-to-roll manufacturing^13^, have been developed to produce large-area graphene sheets. However, the ability to create reliably sized nanopores in the material using scalable techniques is unavailable. Approaches including ion bombardment^10^, e-beam lithography^14^, and ultraviolet-induced oxidative etching^15^ are able to attain the necessary pore-size (that is, <1 nm) for effective separations of small molecules (for example, water, hydrated salt ions, CO_2_, CH_4_ and so on) but remain challenging to scale for the large-area membranes required in industrial applications. Other approaches such as block-copolymer lithography^16^,^17^ may scale to large-area production but cannot achieve small enough pores. Accordingly, exploration of alternative one-atom-thick materials with scalable manufacturability and controllable, suitably sized nanopores is of great interest.

Reduced graphene oxide (rGO), a cousin of graphene, is formed when oxygen atoms are removed from graphene oxide (GO) via either thermal or chemical reduction^18^. Because of the solution-based processing of rGO/GO materials, rGO possesses desirable manufacturability for large-scale production. To date, rGO materials have drawn great attention primarily for their potential as an alternative material to pristine graphene in electronic-related applications. The potential of rGO materials as ultrathin-film membranes, however, has been largely overlooked. Yet, the intrinsic defects in rGO resulting from the removal of carbon-containing by-products such as CO_2_ during the reduction process render rGO a highly promising ultrathin-film membrane. These defects can potentially serve as nanopores to provide selective separations via size exclusion. In order to achieve high performance in a specific separation, a controllable size of nanopores in rGO membranes is essential and a better understanding of the formation of nanopores is the goal of our present study.

In this study, we establish a detailed connection between synthesis parameters and defect sizes in rGO. Moreover, the potential of rGO as ultrathin-film nanoporous membranes in water desalination and natural gas purification applications is demonstrated.

## Results

### rGO structure formation

**Figure 1:**
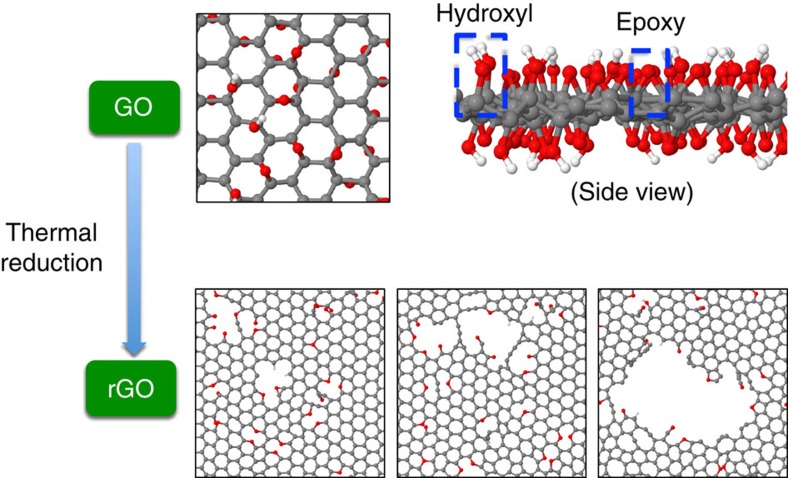
Schematic of the rGO formation. Graphene oxide (GO) is used as a starting material for the preparation of reduced graphene oxide (rGO). Epoxy and hydroxyl functional groups are randomly distributed on both sides of the starting GO sheet. Via a thermal annealing process (that is, thermal reduction), some of atoms are removed, resulting in nanopores formed in rGO materials with a variety of sizes. All structures are represented as ball and stick with carbon, oxygen and hydrogen atoms in grey, red and white colour, respectively.

The production of rGO materials is a complex and multi-dimensional process, which involves various synthesis parameters including initial GO oxygen concentration, initial GO functional group composition (that is, epoxy: hydroxyl ratio) and reduction temperature. Depending on the reduction synthesis parameters, a wide range of nanopore sizes in rGO materials can be achieved (see Fig. 1 for details), which allows rGO to be potentially applicable to a number of separation applications. To effectively and efficiently understand the formation of defects in rGO from reducing GO sheets, state-of-the-art molecular dynamics (MD) simulations using reactive force fields (ReaxFF)^19^ were employed. ReaxFF has been used previously to study the structural evolution of rGO/GO materials at different temperatures, and qualitative agreement with experiments was shown^20^. In this work, we explored the materials space of rGO with the aim of establishing an insightful connection between synthesis parameters and the size of nanopores in rGO. This connection can be used to facilitate the creation of controllable nanopores in rGO materials for a specific application. It is important to note that because of the limitations of simulation timescale, simulation length scale and the accuracy of force fields, the structural predictions based on MD simulations carried out herein should be considered qualitative as opposed to quantitative in nature. More details regarding the computational approach can be found in Methods section.

**Figure 2:**
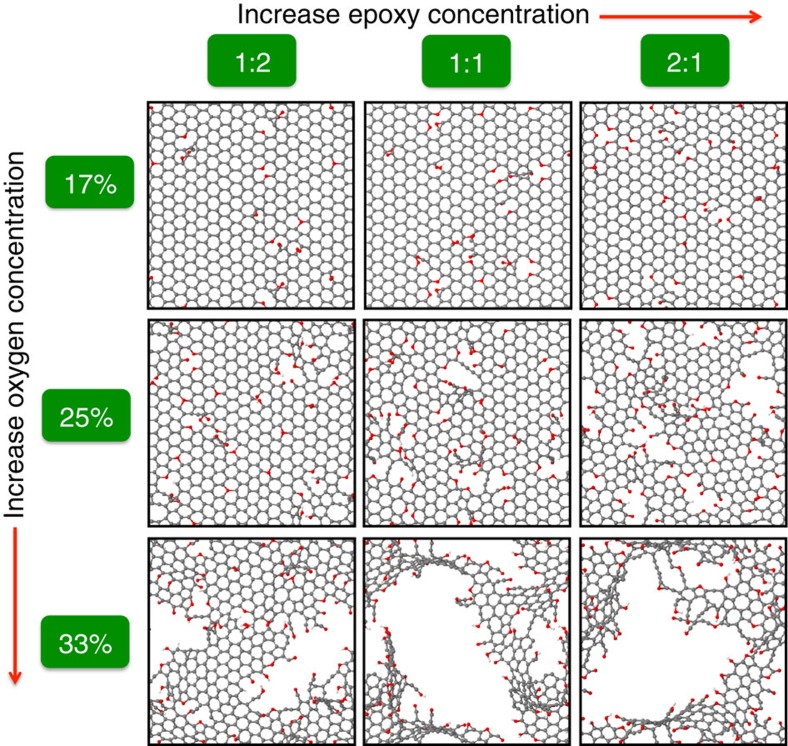
Relationship between defect sizes and synthesis parameters. Representative rGO structures in our simulations obtained from different synthesis conditions are shown in a 3 by 3 matrix. The epoxy/hydroxyl ratio and initial oxygen concentration of GO sheets vary along the horizontal and vertical direction, respectively. The reduction temperature for the formation of these structures was set to 2,500 K. All structures are represented as ball and stick with carbon, oxygen and hydrogen atoms in grey, red and white colour, respectively.

**Figure 3:**
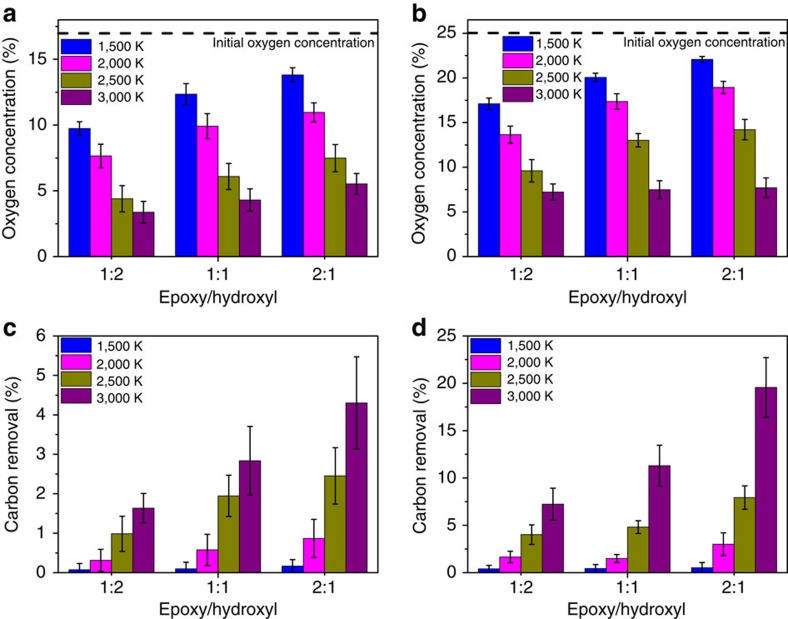
Extent of reduction and carbon removal. The statistically averaged oxygen concentration of resulting rGO materials (**a**,**b**) and the corresponding percentage of carbon removal (**c**,**d**) were calculated over ten different samples for each combination of synthesis parameters. Figures **a**, **c** and **b**, **d** represent the result of initial GO oxygen concentration of 17% and 25%, respectively. The initial oxygen concentration is highlighted using a dashed black line in **a**,**b**. The result of 33% initial oxygen concentration can be seen in Supplementary Fig. 1.

Figure 2 illustrates the effect of total oxygen concentration and functional group ratio of initial GO sheets on the size of nanopores formed in rGO materials at a given reduction temperature. We found that the size of nanopores is strongly influenced by these two variables and may be potentially controlled. GO materials with a higher epoxy: hydroxyl ratio at a given total oxygen concentration generally tend to form larger nanopores in the resulting rGO structure. As shown in Fig. 3a,b, although the extent of reduction (that is, the difference in the oxygen concentration between the initial GO sheet and the final rGO structure) is lower for higher epoxy/hydroxyl ratio, the carbon removal percentage (see Fig. 3c,d for details) is substantially increased. This effect may be attributed to the strain introduced by epoxy functional groups on the structure. The formation of carbon-containing by-products such as CO_2_ and/or CO needs to overcome a high-energy barrier, and the epoxy group imposes strain on the structure^20^, which effectively lowers the barrier and thus promotes carbon removal during the reduction process. A structure with larger nanopores is therefore achieved in the presence of more epoxy groups. Similarly, a starting GO sheet with a higher total initial oxygen concentration was found to have the tendency to result in larger nanopores. This observation is also reflected in Fig. 3c,d, showing that the carbon removal percentage is increased with higher oxygen concentration. Moreover, reduction temperature also plays a crucial role in the formation of nanopores. Structures at a higher temperature possess larger kinetic energies, which increases the likelihood of overcoming reaction barriers to by-product formation. As expected, a higher temperature leads to a higher extent of reduction and, more importantly, facilitates carbon removal to create bigger nanopores in rGO materials (see Fig. 3 and Supplementary Fig. 2 for details). These relationships between the size of nanopores in rGO membranes and synthesis parameters can act as a guide to facilitate the experimental synthesis of rGO membranes with controllable nanopore sizes for a given application.

### rGO membranes for separations

We have shown how the pore sizes of rGO membranes can possibly be controlled via tuning synthesis parameters. It remains, however, crucial to further investigate the performance of rGO as a separation membrane. Specifically, we consider the applications of rGO in water desalination and natural gas purification in this study. Clean water has become increasingly limited, and desalination would represent an ideal solution to supply clean water if not for its high-energy footprint and capital costs. In addition, the removal of CO_2_ from natural gas feed (that is, primarily a CO_2_/CH_4_ mixture) plays an important role in the production of natural gas in order to enhance the heating value of the gas and to meet the requirement of pipeline transportation as pre-conditioned gas can contain as much as 70% CO_2_ by volume^21^. Currently, state-of-the-art amine scrubbing has been widely used for this separation, although substantial energy is required for the solvent regeneration^22^. To understand the separation performance of rGO membranes in these two applications, MD simulations were used to predict permeate fluxes and selectivity (see Methods section for details).

Table 1 summarizes the averaged water permeability and salt rejection of rGO obtained from different synthesis parameters in desalination applications. For a starting GO sheet with a low initial oxygen concentration (17%), the pore size of resulting rGO membranes is too small to allow passage of any water molecules. At a higher oxygen concentration (25%), the size of nanopores in rGO can be sufficiently large to allow water passage only at a higher reduction temperature (≥2,500 K) and larger epoxy/hydroxyl ratio (≥1). Synthesis conditions with a too high functional group ratio and temperature lead to nanopores large enough to allow both water molecules and salt ions to pass through the membrane, resulting in an ineffective RO process. At an oxygen concentration of 33%, a lower reduction temperature and smaller functional group ratio are preferred. These results reflect the relationship between nanopore sizes and synthesis parameters obtained previously, and Table 1 shows that, under properly chosen synthesis conditions, the resulting rGO membranes can possess an order of magnitude higher water permeability compared with current state-of-the-art polyamide-based RO membranes while excellent ability to reject salt is still maintained.

Interestingly, although a better desalination performance using rGO membranes may be expected if rGO synthesis parameters are further optimized, compared with nanoporous graphene^8^ rGO achieves roughly an order of magnitude lower permeability. This difference arises from the non-uniform size of nanopores formed in rGO membranes, leading to an important consideration in the design of rGO membranes. As shown in Supplementary Fig. 3, a range of pore sizes is achieved even under a given synthesis condition. It is therefore important to ensure the largest pore created after reduction processes does not allow the passage of salt ions. Consequently, the performance of rGO membranes in general cannot be as optimal as nanoporous graphene with a uniform pore size. However, we note that a recent study showed that, beyond a factor of roughly three in membrane permeability compared with currently available RO membranes, further enhancement makes only a marginal improvement on the energy efficiency and cost reduction of a desalination process^4^.

To better quantify the pore size distribution of rGO, we calculate the largest free sphere radius of each membrane based upon its projected atomic coordinates on the *X*–*Y* plane, representing an effectively minimum size of the largest pore of an rGO structure. The largest pore radius in rGO from GO with initial oxygen concentrations of 17% and 25% is summarized in Supplementary Tables 1 and 2, respectively. In agreement with the observed rGO desalination performance shown in Table 1, rGO formed from a too low initial GO oxygen concentration (17%) does not possess sufficiently large pores for water filtration. The effects of synthesis parameters on the rGO pore size distribution are shown in Supplementary Fig. 4. Our analysis indicates that synthesis parameters with a high reduction temperature and a large epoxy/hydroxyl ratio are likely to not only result in big pores but also lead to a broad pore size distribution. Under these conditions, accurate control of pore size in rGO may be challenging and require fine-tuning of the synthesis parameters.

**Figure 4:**
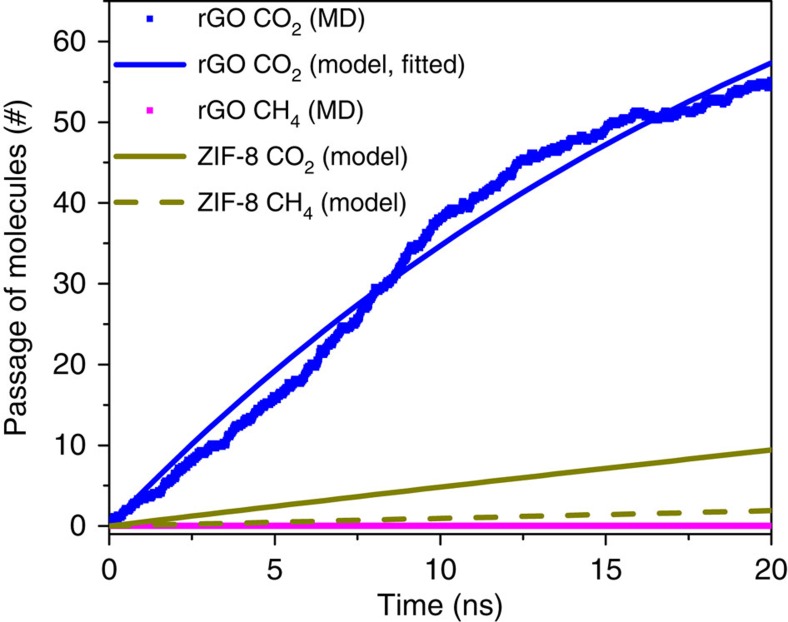
Comparison between rGO membrane and ZIF-8 membrane for CO_2_/CH_4_ separation. Molecular simulations were carried out to estimate the number of gas molecules passing through the rGO membrane as a function of simulation time averaged over five independent simulations (CO_2_ and CH_4_ are represented as blue and magenta squares, respectively). The CO_2_ result obtained from MD simulations was further fitted using the membrane process model (see Methods section), and the CO_2_ permeance of the rGO membrane was determined to be 2.1 × 10^−4^ (mol m^−2^ s^−1^ Pa^−1^; solid blue line). As a comparison, the corresponding CO_2_ (solid dark yellow line) and CH_4_ (dashed dark yellow line) results using ZIF-8 membranes are also provided, which were estimated using the membrane process model (see Methods section) with experimentally reported parameters^23^.

We also explore the use of rGO membranes in natural gas purification. Here we select a resulting rGO membrane from our synthesis calculations (see Supplementary Fig. 5 for details) to study its purification performance computationally. The statistically averaged gas permeation of the membrane as a function of time for CO_2_ and CH_4_ molecules is shown in Fig. 4. The result shows the membrane only allows the passage of CO_2_ molecules while blocking CH_4_ molecules due to size exclusion, demonstrating opportunities for effective natural gas purification. Furthermore, compared with the performance of ZIF-8 (ref. 23), one of the most promising membranes for CO_2_/CH_4_ separation to date, the rGO membrane possesses a highly enhanced permeate flux. Further comparisons between the rGO membrane and other materials reported in the literature such as zeolites can be found in Supplementary Table 3, and in all cases, rGO membranes show promise for this type of purification. It should be noted that an effective CO_2_/CH_4_ separation is very difficult as the difference between their kinetic diameter is merely ∼0.5 Å. Accurate control over the pore formation in rGO is therefore essential. Supplementary Fig. 6 indicates that further optimization on rGO synthesis parameters is needed for this separation as we found an outlier rGO from the same synthesis conditions of the previously selected rGO membrane has a marginally too large pore, allowing fast passage of CH_4_ molecules. With the previously established relationship between synthesis parameters and defect sizes, it is suggested that one needs to slightly decrease the reduction temperature, initial oxygen concentration and/or epoxy/hydroxyl ratio to ensure the obtained rGO membranes possess pores that are diffusively limited to CH_4_ molecules.

Although this work focuses mainly on monolayer rGO membranes for their separation performance, a realistic membrane might consist of one to few rGO layers. Recent work has shown that the interlayer regions between a multilayered, stacked GO membrane can allow for fast water permeation, which indicates the interlayer regions might not create a substantial barrier for water transport, depending on the channels formed between layers^24^,^25^,^26^. In addition, the interlayer space has been found to facilitate selective permeation^27^,^28^. Finally, a multilayered structure is expected to enhance the membranes' mechanical stability compared with a monolayer one. We have computationally studied the mechanical strength of a promising desalination rGO membrane obtained in this work using a biaxial strain test. Our calculations show that the fracture stress of the rGO structure is roughly 40 GPa, about 35% of the value obtained for a nanoporous graphene membrane using a similar approach^29^. A future study is, however, needed to understand the design of corresponding supporting substrates as well as extensively investigate the mechanical properties of rGO as a function of synthesis parameters.

## Discussion

We made use of state-of-the-art molecular simulations to understand the formation of nanopores in rGO materials and investigate their performance in separation applications, leading to insights into the synthesis of promising rGO membranes. In this work, a large rGO materials space was explored computationally by considering a wide range of synthesis parameters and taking into account the amorphous nature of GO. We note that additional factors in the experimental synthesis such as the multi-layered nature of GO structures, the role of an amorphous surface layer, and boundary effects were not considered in the calculations; however, our results provide qualitative information towards the formation of nanopores in rGO, and critically, a demonstration that under suitable processing conditions controlled pore formation is achievable. It was found that initial GO sheets with a smaller oxygen concentration and lower ratio of epoxy/hydroxyl will require a higher reduction temperature to achieve a given size of nanopores. A sufficiently large oxygen concentration is, however, needed otherwise a reasonably sized nanopore for separations cannot be obtained. These results can provide useful guidelines for experimental rGO synthesis, facilitating the creation of controllable nanopores in the material. The corresponding separation performance of resulting rGO membranes in water desalination and CO_2_ removal from natural gas was also studied. Our computational predictions have demonstrated the potential of rGO membranes in these applications, if synthesized under properly chosen conditions. We anticipate that the present study can push the development of ultrathin-film nanoporous membranes using rGO materials forward for separation applications.

## Methods

### Molecular simulations

Molecular simulations performed in this work were carried out within the LAMMPS package^30^ to study the formation of rGO materials and investigate their separation performance. Details regarding simulations are given below.

### rGO structure formation

MD simulations were employed to simulate the formation of rGO materials from thermally reducing GO sheets^20^,^31^. To properly describe the bond association/disassociation events during the reduction process, ReaxFF^19^ potentials were adopted. Initial, starting GO sheets have a dimension of approximately 3.4 nm by 3.2 nm (the *X*–*Y* plane), and epoxy and hydroxyl functional groups were randomly distributed on both sides of the sheet. Along the direction that is perpendicular to the GO sheet (the Z-direction), the simulation box has a dimension of 15 nm, which allows the simulation to properly take the surface adsorption of by-product molecules such as H_2_O and CO_2_ and their reaction with the rGO sheet into account during the reduction process. Periodic boundary conditions were applied on the *X*, *Y* and *Z* directions. Structural optimization of the initial GO materials using ReaxFF was performed before the reduction process. The ReaxFF-optimized GO sheet was then heated to the designed reduction temperature at a rate of ∼6 K fs^−1^ from 10 K. The GO sheet was annealed at the reduction temperature for 400 ps, which was found to be sufficiently long to ensure the formed rGO structure has been stabilized (see Supplementary Figs 7 and 8 for details). A Berendsen thermostat was used to control the system's temperature with a damping factor of 100 time steps. In this study, the MD time steps of 0.1, 0.08, 0.05 and 0.05 fs were adopted for the reduction temperatures of 1,500, 2,000, 2,500 and 3,000 K, respectively, which were found to be sufficiently small to ensure energy conservation (see Supplementary Fig. 9 for details). During the reduction processes, to mimic the experimental reduction environment against vacuum, molecular by-products released from the GO sheets were removed periodically every 200 fs when these molecules diffuse away from the GO sheet at a distance of more than 5 nm along the *Z*-direction. The final rGO structure from each simulation was further examined based on bonding connectivity (that is, use 0.175 nm as the cutoff distance to determine the existence of chemical bonds between two atoms) to remove any remaining by-products adsorbed at the rGO surface, leaving only a pure rGO sheet. This study considers three synthesis parameters: (i) initial oxygen concentration (17, 25, and 33%) of GO materials, (ii) initial epoxy/hydroxyl ratio (1:2, 1:1, and 2:1) of GO materials and (iii) reduction temperatures (1,500, 2,000, 2,500, and 3,000 K). As GO (rGO) is an amorphous material, a representatively large number of initial GO samples for each synthesis condition needs to be considered. In this study, ten different samples with randomly distributed configurations of functional groups were used for each combination of oxygen concentration and functional group ratio. In total, 360 rGO membranes were obtained in this work. Here we note that in order to study rGO formation within the time scale of MD simulations (< 1 ns), higher temperatures (1,500–3,000 K) compared with typical GO reduction have been employed in this work. Although this renders quantitative comparisons of temperature unreasonable, qualitative trends of structure evolution with temperature have been found to be in good agreement with experimental measurements^20^.

### Desalination simulation

Desalination performance of each resulting rGO membrane was studied using MD simulations. To allow the rGO membrane to be fully flexible during the simulation, ReaxFF was adopted to describe the rGO membrane, whereas a Lennard-Jones (L-J) plus Coulomb potential was used to describe the interactions of salt water–salt water and salt water–rGO membrane. For the non-ReaxFF interactions, the SPC/E model^32^ was adopted for water molecules with the corresponding parameters proposed by Joung *et al.* for salt ions^33^. In addition, the Dreiding force field^34^ was used to assign L-J parameters for C, O and H elements in rGO materials. For all pair-wise terms, this work made use of Lorentz-Berthelot mixing rule.

The simulation box consists of an rGO membrane, a piston and salt water. The salt water in the feed side has 16 Na^+^ and 16 Cl^−^ ions solvated by 900 water molecules. The simulations were performed at 300 K using a Nosé–Hoover thermostat with a damping factor of 100 time steps. The simulation time step was chosen to be 0.3 fs with a total simulation time of ∼1 ns. The applied pressure on the piston was set to be ∼125 MPa, which is higher than the typical value used in a RO plant, in order to obtain better statistics for the short time scales involved in these MD simulations^8^.

### Gas separation simulation and modelling

Gas separation performance was also investigated using MD simulations. Compared with the desalination simulations, a much lower pressure condition is considered. Accordingly, rGO membranes were assumed to be rigid in these simulations. Classical 12-6 Lennard-Jones and Coulomb interactions were used to describe intermolecular interactions. TraPPE force fields^35^ were adopted for CH_4_ and CO_2_ molecules, whereas the Dreiding force field^34^ was used to assign Lennard-Jones parameters for rGO atoms. The Lorentz-Berthelot mixing rule was used for all pair-wise terms. The charge equilibration (QEq) model^36^ was used to determine the atomic charges in rGO materials.

The simulation box has a total dimension of 40 nm along the *Z*-direction (perpendicular to the rGO membrane), which consists of two compartments with an rGO membrane located in between. For a given gas component of interest, CO_2_ or CH_4_, 200 molecules were initially placed in one compartment (this acts as the feed or retentate side). MD simulations were carried in the canonical (NVT) ensemble with a Nosé–Hoover thermostat at a damping factor of 100 time steps, that is, 100 fs, for a total of 20 ns. The number of gas molecules on the feed side was recorded as a function of simulation time. Five independent simulations were carried out in order to obtain good statistics.

To estimate the gas permeance of membranes from MD simulations and compare the performance with other materials reported in the literature, a macroscopic process model was built based upon a typical membrane governing equation:


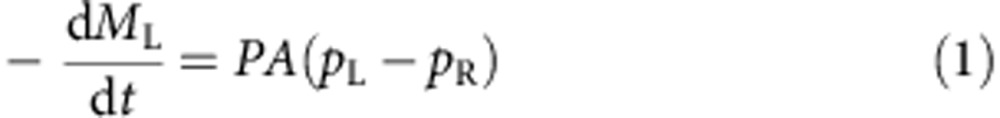


where *M*_L_ is the total moles of gas on the feed side, *p* is the gas permeance, *A* is the membrane area, and *p*_L_ and *p*_R_ are the pressure in the feed and permeate sides, respectively. In this model, the pressure of each compartment was estimated by using a Peng–Robinson equation of state. Finally, the gas permeance, *P*, of a given gas component can be obtained by fitting the above equation to the number of gas molecules on the feed side as a function of time obtained from MD simulations.

## Additional information

**How to cite this article:** Lin, L.-C. and Grossman, J. C. Atomistic understandings of reduced graphene oxide as an ultrathin-film nanoporous membrane for separations. *Nat. Commun.* 6:8335 doi: 10.1038/ncomms9335 (2015).

## Supplementary Material

Supplementary Information Supplementary Figure 1-9, Supplementary Tables 1-3 and Supplementary References

## Figures and Tables

**Table 1:** Separation performance of rGO membranes in water desalination.

	**Water flux (L** **cm**^−2^ **day**^−1^ **MPa**^−1^)			**Salt rejection (%)**		
**Initial oxygen concentration**	1:2*	1:1*	2:1*	1:2*	1:1*	2:1*
*T=1,500 K*						
17%	0.0	0.0	0.0	100	100	100
25%	0.0	0.0	0.0	100	100	100
33%	0.0	**2.6**	**2.0**	100	**99**	**100**
						
*T=2,000 K*						
17%	0.0	0.0	0.0	100	100	100
25%	0.0	0.0	0.1	100	100	100
33%	**2.1**	**15.3**	(L)	**100**	**99**	(L)
						
*T=2,500 K*						
17%	0.0	0.0	0.0	100	100	100
25%	0.0	0.0	15.4	100	100	93
33%	27.4	(L)	(L)	94	(L)	(L)
						
*T=3,000 K*						
17%	0.0	0.0	0.0	100	100	100
25%	0.1	**3.0**	(L)	100	**100**	(L)
33%	(L)	(L)	(L)	(L)	(L)	(L)

rGO, reduced graphene oxide.

*Initial epoxy/hydroxyl ratio.

Averaged water flux and salt rejection obtained from MD simulations are summarized. The salt rejection is defined as the percentage of salt ions staying in the retentate or feed side of the desalination process after a simulation time of ∼1 ns. Those combinations of synthesis parameters that result in promising desalination performance are highlighted in bold. The letter ‘(L)' indicates that nanopores in rGO membranes are too large and both the water molecules and salt ions can freely pass through the rGO membrane (that is, unable to achieve an effective reverse osmosis process).
