# Supplementary material for: Atomistic understandings of reduced graphene oxide as an ultrathin-film nanoporous membrane for separations
Source: Nat Commun. 2015 Sep 23;6:8335. doi: 10.1038/ncomms9335 (PMC4667428; doi:10.1038/ncomms9335)
Supplement: Supplementary Information — Supplementary Figure 1-9, Supplementary Tables 1-3 and Supplementary References [file ncomms9335-s1.pdf]

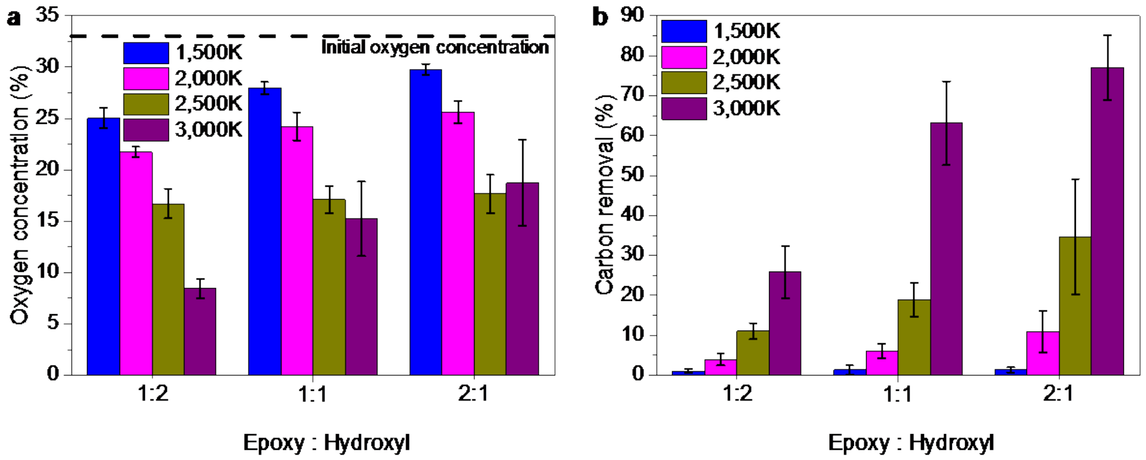

Supplementary Figure 1 Extent of reduction and carbon removal. The (a) statistically averaged oxygen concentration of resulting rGO structures and the (b) corresponding percentage of carbon removal were calculated over ten different samples for each combination of synthesis parameters. The figure shows the result of initial GO materials with an oxygen concentration of 33% that is highlighted using a dashed black line in (a).

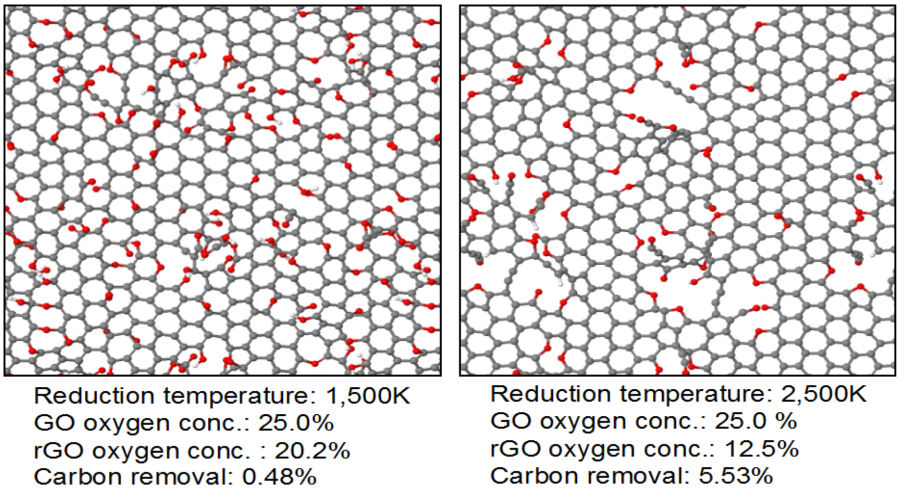

Supplementary Figure 2 The effect of reduction temperatures on the pore formation of rGO. The annealing temperatures used for the left and right structures were set to be 1500K and 2500K, respectively. With a higher temperature, the extent of reduction is increased and, most importantly, the carbon removal percentage is enhanced. As a result, a larger defect is observed in the right structure. In these simulations, the initial oxygen concentration and epoxy: hydroxyl ratio of GO materials were chosen to be 25% and 1:1, respectively. These structures are represented as stick and ball with carbon, oxygen, and hydrogen atoms in grey, red, and white color, respectively.

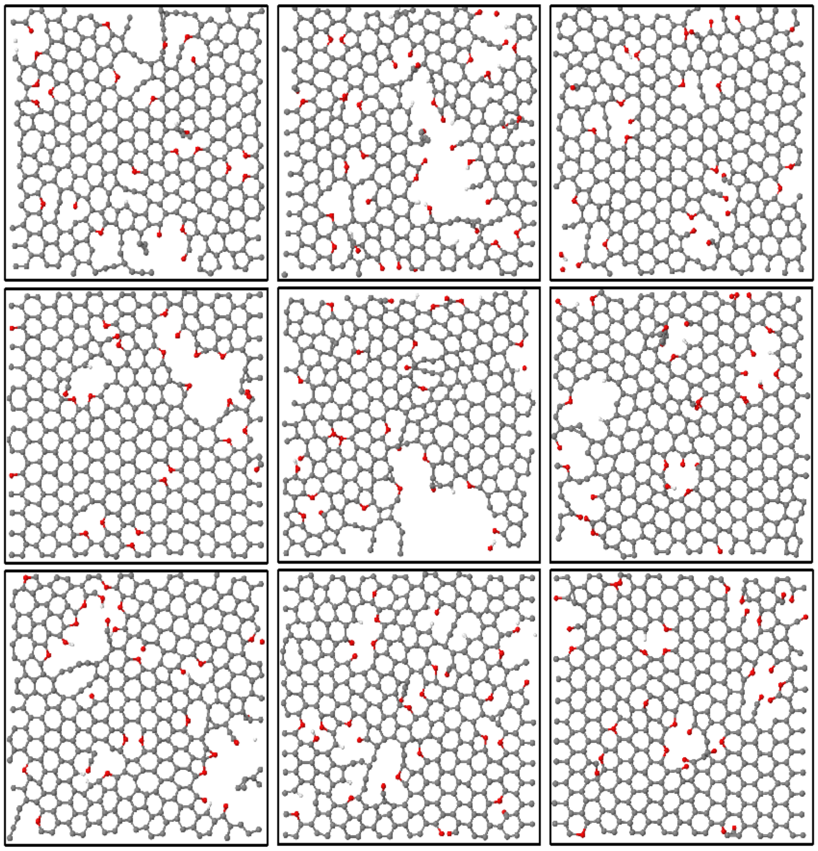

Supplementary Figure 3 Non uniform pore sizes of rGO membranes. Different structural features (i.e., pore sizes) were obtained at a total oxygen concentration of 25%, epoxy: hydroxyl ratio of 1:1, and temperature of 3000K. These structures are represented as stick and ball with carbon, oxygen, and hydrogen atoms in grey, red, and white color, respectively. A unit cell at a dimension of about 3.4 nm by 3.2 nm of each structure is shown in this figure.

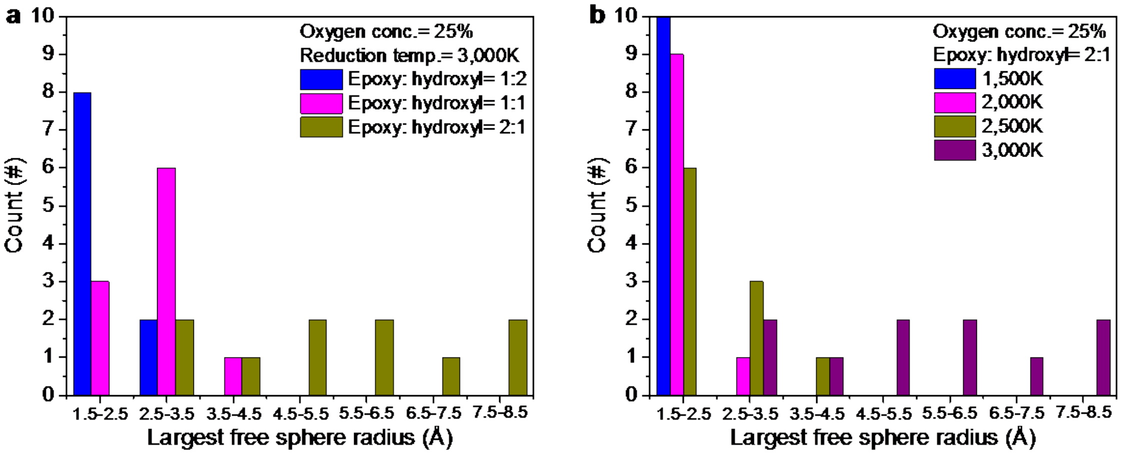

Supplementary Figure 4 rGO pore size distribution. The effects of (a) epoxy: hydroxyl ratios and (b) reduction temperatures on the rGO largest free sphere radius distribution are shown in this figure. The largest free sphere radius of each rGO membrane was computed based upon the membrane's projected coordinates on the X-Y plane, representing an effectively minimum radius of the largest pore of an rGO membrane. The regions within the van der Waals surface of each atom were not excluded.

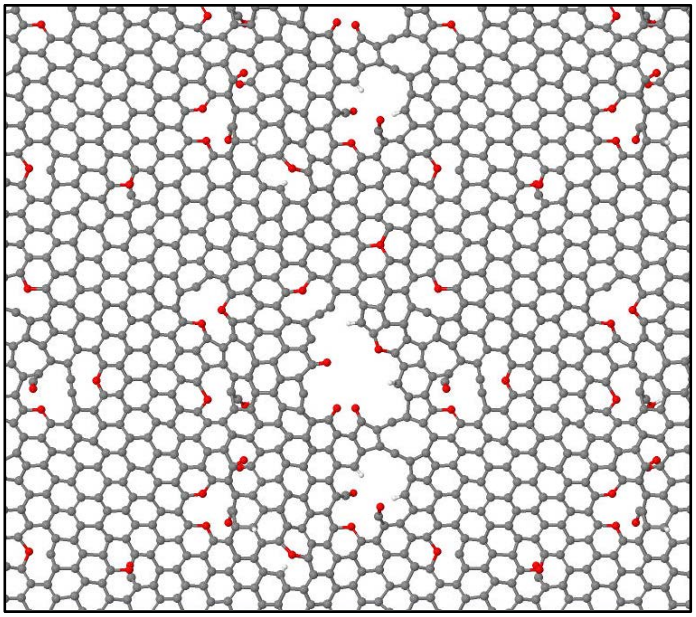

Supplementary Figure 5 Atomic structure of the rGO membrane used in this work for CO_2/CH_4 separation. The illustrated structure is one of the resulting rGO materials obtained from the synthesis parameters using an initial oxygen concentration of 25%, epoxy: hydroxyl ratio of 1:2, and reduction temperature of 3000K (i.e., denoted as S5 that is listed in Supplementary Table 2 under the aforementioned condition). The structure is represented as stick and ball with carbon, oxygen, and hydrogen atoms in grey, red, and white color, respectively.

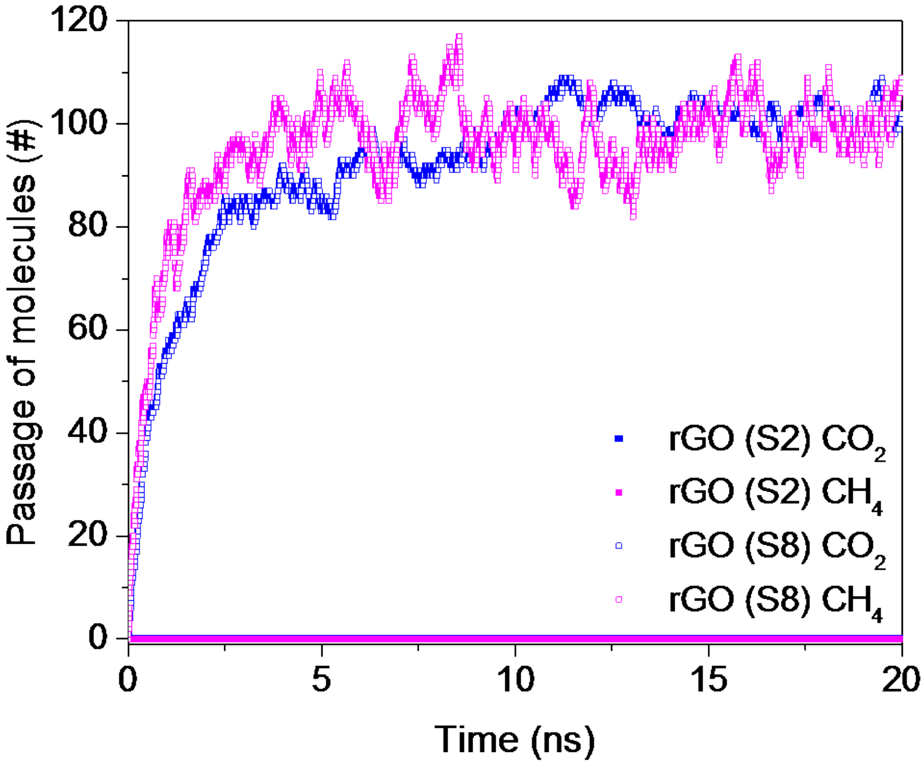

Supplementary Figure 6 rGO membranes for CO_2/CH_4 separation. This figure shows the passage of CO_2 and CH_4 molecules through rGO membranes (i.e., denoted as S2 and S8 that are listed in Supplementary Table 2 under the following condition) obtained from reducing GO with an initial oxygen concentration of 25% and epoxy: hydroxyl ratio of 1:2 at 3000K as a function of time from a single MD simulation. CO_2 and CH_4 are represented as blue and magenta squares, respectively.

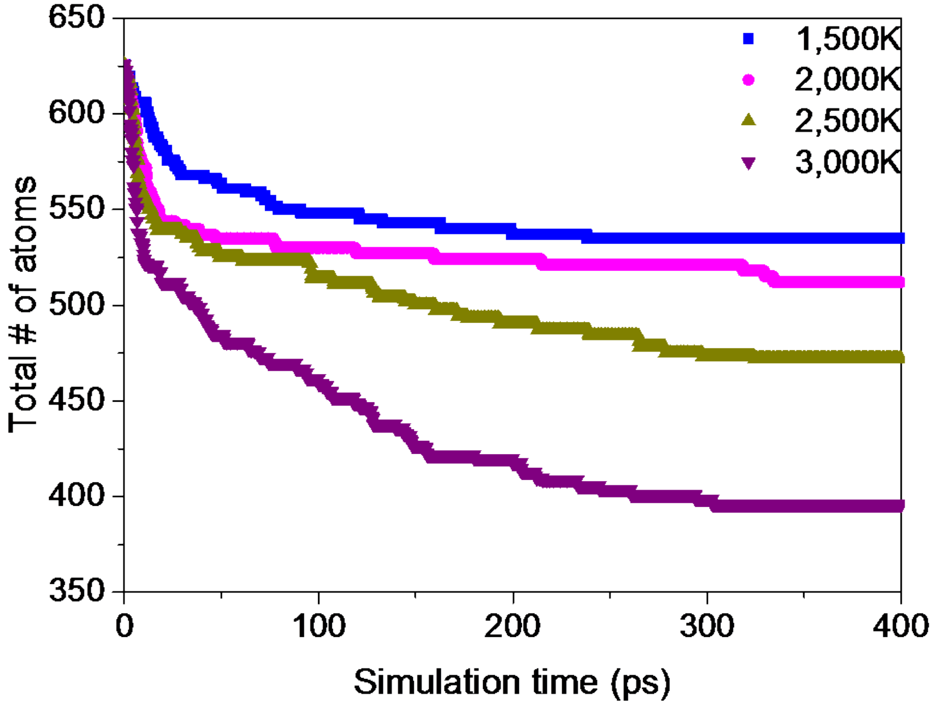

Supplementary Figure 7 Simulation time for the formation of rGO. A total simulation time of 400 ps was found to be sufficiently long for reduction processes at temperatures ranging from 1500K to 3000 K. As can be seen from the figure, the total number of atoms in rGO materials remains constant after roughly 300 ps. In these simulations, the initial oxygen concentration and epoxy: hydroxyl ratio of GO materials were chosen to be 25% and 1:1, respectively.

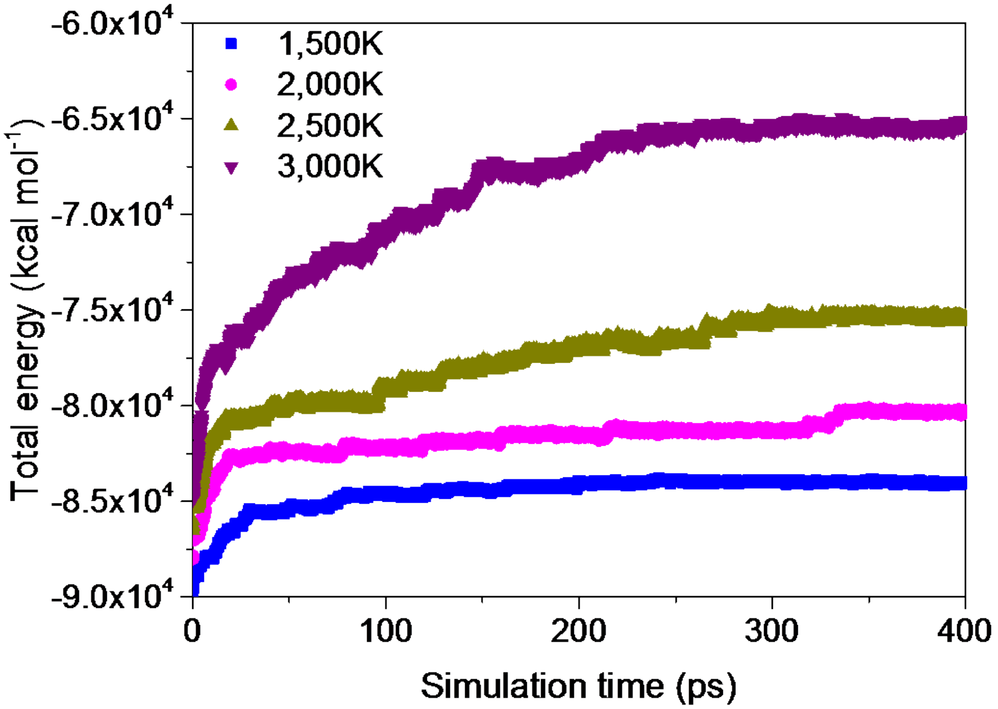

Supplementary Figure 8 Simulation time for the formation of rGO. A total simulation time of 400 ps was found to be sufficiently long for reduction processes at temperatures ranging from 1500K to 3000 K. As shown in the figure, the total energy of simulated systems reaches equilibrium after roughly about 300 ps. In these simulations, the initial oxygen concentration and epoxy: hydroxyl ratio of GO materials were chosen to be 25% and 1:1, respectively.

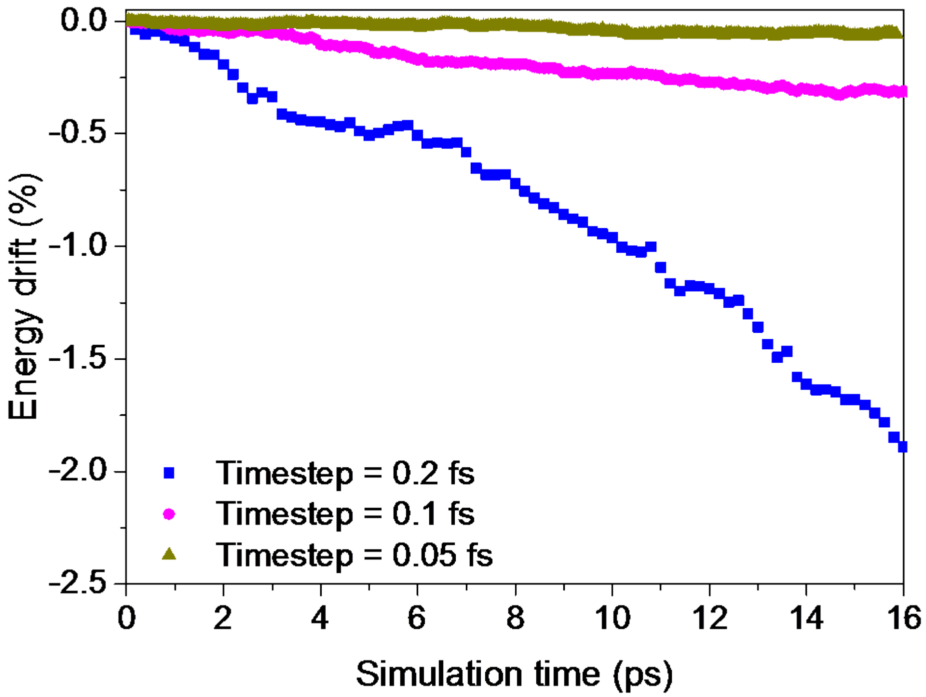

Supplementary Figure 9 Simulation time step used for the formation of rGO at 2500K. A time step of 0.05 fs is needed at the reduction temperature of 2500K in order to maintain energy conservation in a NVE ensemble. In these simulations, the initial oxygen concentration and epoxy: hydroxyl ratio of GO materials were chosen to be 25% and 1:1, respectively.

Supplementary Table 1 Largest free sphere radius. The largest free sphere radius of each rGO formed from reducing GO with an initial oxygen concentration of 17% is listed. The largest sphere radius of a given membrane was calculated based on its projected atomic coordinates on the X-Y plane and the regions inside the van der Waals surface of each atom were not excluded. Considering rGO membranes are not perfectly planar, the determined radius of the largest pore of an rGO may be underestimated.

Sample	Epoxy: hydroxyl= 1:2	Epoxy: hydroxyl= 1:1	Epoxy: hydroxyl= 2:1
		T=1,500K	
S1	1.4119	1.5538	1.4295
S2	1.4546	1.4184	1.5789
S3	1.4446	1.4532	1.5115
S4	1.6382	1.4960	1.4280
S5	1.4564	1.4369	1.4433
S6	1.4661	1.4401	1.4861
S7	1.4263	1.4170	1.5346
S8	1.4972	1.4472	1.4545
S9	1.4204	1.4293	1.5137
S10	1.4321	1.5007	1.6889
Sample		T=2,000K	
S1	1.4470	1.5934	1.4840
S2	1.4698	1.4766	1.4846
S3	1.4353	1.4085	1.4808
S4	1.4391	1.5064	1.4516
S5	1.4375	1.4375	1.4934
S6	1.4320	1.4366	1.5428
S7	1.4699	1.4607	1.4817
S8	1.4678	1.4154	1.6721
S9	1.4226	1.5360	1.5766
S10	1.4342	1.4741	1.5439
Sample		T=2,500K	
S1	1.4159	1.5060	1.7106
S2	1.4397	1.4913	1.4566
S3	1.4596	1.7381	1.4645
S4	1.4143	1.4259	1.4496
S5	1.4410	1.4089	1.5469
S6	1.4383	1.4795	1.4831
S7	1.4366	1.4291	1.7923
S8	1.6497	1.5389	1.4950
S9	1.4922	1.5128	1.5323
S10	1.4203	1.4177	1.4287
Sample		T=3,000K	
S1	1.4082	1.7728	2.6121
S2	1.4547	1.4896	1.4369
S3	1.4150	1.7596	1.5199
S4	1.4050	1.4851	1.4155
S5	1.4746	1.4728	1.6556
S6	1.4357	1.4032	1.5477
S7	1.7354	1.6117	1.4183
S8	1.5656	1.5317	1.6354
S9	1.5326	1.4219	1.7120
S10	1.8617	1.8080	1.5644

Supplementary Table 2 Largest free sphere radius. The largest free sphere radius of each rGO formed from reducing GO with an initial oxygen concentration of 25% is listed. The largest sphere radius of a given membrane was calculated based on its projected atomic coordinates on the X-Y plane and the regions inside the van der Waals surface of each atom were not excluded. Considering rGO membranes are not perfectly planar, the determined radius of the largest pore of an rGO may be underestimated.

Sample	Epoxy: hydroxyl= 1:2	Epoxy: hydroxyl= 1:1	Epoxy: hydroxyl= 2:1
		T=1,500K	
S1	1.5233	1.5753	1.6275
S2	1.4774	1.6169	2.0577
S3	1.6098	1.6023	1.5537
S4	1.6639	1.6489	1.6936
S5	1.4428	1.6141	1.7385
S6	1.4451	1.4700	1.6018
S7	1.4549	1.4813	1.6714
S8	1.4265	1.6005	1.7330
S9	1.4837	1.6016	1.5428
S10	1.4275	1.7063	2.0277
Sample		T=2,000K	
S1	1.8896	1.6518	1.7707
S2	1.5141	2.6063	1.8199
S3	1.5599	1.9828	2.0134
S4	1.4894	1.7430	3.1744
S5	1.6458	1.5856	2.2855
S6	1.4747	1.8179	2.0761
S7	1.4385	1.8621	1.9125
S8	1.5571	1.5738	1.8821
S9	1.7763	1.5413	2.2563
S10	1.5668	1.6278	2.2293
Sample		T=2,500K	
S1	1.5354	2.0669	4.8118
S2	1.5958	1.9411	2.9551
S3	1.7613	2.2182	2.9876
S4	1.4263	1.7398	2.1793
S5	1.5762	2.2767	2.0714
S6	1.5358	1.5430	3.2844
S7	1.5186	1.9509	2.3303
S8	1.4474	2.2805	2.0446
S9	1.9419	2.0064	2.3293
S10	1.5938	2.1279	2.3100
Sample		T=3,000K	
S1	1.5041	2.5900	4.8760
S2	1.8932	2.1003	2.8281
S3	2.2875	3.3729	6.1525
S4	1.7777	3.1277	4.9301
S5	2.6894	2.6546	6.6958
S6	1.8895	2.6849	3.4886
S7	1.9814	2.0003	5.6319
S8	2.9643	4.5129	4.0726
S9	1.9789	2.0034	8.3770
S10	1.5016	2.8757	8.0664

Supplementary Table 3 Separation performance of rGO membranes in natural gas purification. Comparison of the CO_2/CH_4 separation performance between the chosen rGO membrane (see Supplementary Fig. 5) and several different materials reported in the literature. The gas permeance of the rGO membrane was obtained by fitting to the MD result using the process model described in Methods section (i.e., gas permeance is the fitted parameter).

Materials	CO_2 permeance (mol m^{-2} s^{-1} Pa^{-1})	CO_2/CH_4 selectivity
rGO (this work, sim.)	2.1×10^{-4}	INF
Bio-MOF-1 (exp.)[1]	$<1.2 \times 10^{-6}$	< 2.6
ZIF-8 (exp.)[2]	$<2.4 \times 10^{-5}$	<7.0
DDR (exp.)[3]	7×10^{-8}	280
MFI (exp.)[4]	$<7 \times 10^{-6}$	~ 4
SAPO-34 (exp.)[5]	$<2.0 \times 10^{-6}$	<171

References

[1] Bohrman, J.A. & Carreon, M.A., Synthesis and CO_2/CH_4 separation performance of Bio-MOF-1 membranes. *Chem. Commun.* **48**, 5130–5132 (2012).

[2] Venna, S. & Carreon, M. Highly permeable zeolite imidazolate framework-8 membranes for CO_2/CH_4 separation. *J. Am. Chem. Soc.* **132**, 76–78 (2010).

[3] Tomita, T., Nakayama, K. & Sakai, H. Gas separation characteristics of DDR type zeolite membrane. *Microporous Mesoporous Mater.* **68**, 71–75 (2004).

[4] Sandström, L., Sjöberg, E. & Hedlund, J. Very high flux MFI membrane for CO_2 separation. *J. Membr. Sci.* **380**, 232–240 (2011).

[5] Carreon, M.A., Li, S. & Falconer, J.L. Alumina-supported SAPO-34 membranes for CO_2/CH_4 separation. *J. Am. Chem. Soc.* **130**, 5412–5413 (2008).
